# Dr. Edward L. Groess

**Published:** 1901-07

**Authors:** 


Dr. Edward L. Groess, U. of B., '93, of Buffalo, died at his home, June 19, 1901, from paralysis of the heart. He was born in Buffalo on May 10, 1863, and was a son of John G. Groess, one of the pioneers of the city. The funeral was held from the family home, corner of West and Auburn avenues, at 2 o'clock on Saturday afternoon, June 22. Dr. Groess was unmarried, and is survived by his father, two brothers, George and John J., Jr., and a sister, Mrs. Louisa Talbot.

